# Development of Novel Squid Gladius Biomaterials for Cornea Tissue Engineering

**DOI:** 10.3390/md22120535

**Published:** 2024-11-28

**Authors:** Ingrid Garzón, Juan Muñoz-Hurtado, Juan Pereira-Martínez, Ana M. Ionescu, Juan de la Cruz Cardona, María Tejada-Casado, María del Mar Pérez, Fernando Campos, Jesús Chato-Astrain, Miguel Alaminos

**Affiliations:** 1 Tissue Engineering Group, Department of Histology, Faculty of Medicine, University of Granada, E18016 Granada, Spain; igarzon@ugr.es (I.G.); juanmurtado@correo.ugr.es (J.M.-H.); juanbelmez1@correo.ugr.es (J.P.-M.); malaminos@ugr.es (M.A.); 2 Instituto de Investigación Biosanitaria ibs.GRANADA, E18011 Granada, Spain; anaionescu@ugr.es (A.M.I.); cardona@ugr.es (J.d.l.C.C.); mmperez@ugr.es (M.d.M.P.); 3 Laboratory of Biomaterials Optics, Department of Optics, Faculty of Science, University of Granada, E18071 Granada, Spain

**Keywords:** natural biomaterials, squid, tissue engineering, cornea

## Abstract

Cornea tissue engineering is strictly dependent on the development of biomaterials that fulfill the strict biocompatibility, biomechanical, and optical requirements of this organ. In this work, we generated novel biomaterials from the squid gladius (SG), and their application in cornea tissue engineering was evaluated. Results revealed that the native SG (N-SG) was biocompatible in laboratory animals, although a local inflammatory reaction was driven by the material. Cellularized biomaterials (C-SG) demonstrated that the SG provides an adequate substrate for cell attachment and growth, and corneal epithelial cells cultured on this biomaterial were able to express crystallin alpha, a marker for this type of cells. Biomechanical analyses showed that N-SG biomaterials have higher Young modulus and lower traction deformation than control native corneas (CTR), and C-SG showed a similar Young modulus than CTR. Analysis of the optical properties of these samples revealed that the diffuse transmittance of N-SG and C-SG were higher than CTR, with the diffuse reflectance showing the opposite behavior. These results confirm the putative usefulness of this abundant marine-derived biomaterial that can be obtained as a byproduct of the fishing industry.

## 1. Introduction

Biomaterials are natural or synthetic products that can be used in contact with a biological system or organism for therapeutic or diagnostic purposes [1]. In the field of tissue engineering, biomaterials can be used as scaffolds to sustain cell attachment, growth, and proliferation, thus allowing the generation of different types of tissue substitutes [2]. In this case, biomaterials should fulfill several requirements, including ex vivo and in vivo biocompatibility, biodegradation or bioresorption, among others [3], and should support cell adhesion and proliferation [4].

In contrast to synthetic biomaterials, natural biomaterials typically show high biocompatibility and biodegradability, which may expand their biological applications [5]. Several types of natural biomaterials have been successfully used in the tissue engineering of different tissues and organs, such as the human skin [6], cornea [7] and nerve [8], among others. However, the ideal biomaterial has not been developed to date, and most of the currently available biomaterials are subjected to several drawbacks and disadvantages that include weak biomechanical properties, biofabrication complexity, low biodegradation rate or induction of a pro-inflammatory host response [9]. In the case of the human cornea, another common disadvantage is the lack of transparency of most of the currently available biomaterials.

In the field of cornea tissue engineering, different types of biomaterials have been described, including natural hydrogels, such as collagen [10], chitosan [11] or fibrin [12], synthetic biomaterials [13], such as polyacrylamide (PAAM), polyethylene glycol (PEG), and polylactic acid (PLA) [14], and several types of smart biomaterials able to response to changes in pH, temperature or electric fields, among others [15]. In most cases, human cells obtained from corneas biopsies are cultured on the surface of these biomaterials to obtain a biological substitute of the human cornea, although some cornea models include other cell types immersed within the biomaterial structure to generate a stromal substitute that is then used to support the growth and development of a superficial epithelial layer [16]. In addition to these biological substitutes of the human cornea, several types of keratoprosthesis have been described and used clinically [17], although the results of these inert products are typically suboptimal. In addition, only a few of these biomaterials have been evaluated in patients [18]. Until now, clinical trials have been implemented using artificial corneas based on fibrin [19], fibrin-agarose [20], collagen [21] and decellularized tissues [22], with promising results. However, the search for novel biomaterials that can fulfill all requirements for cornea tissue engineering is still an ongoing need.

Biomaterials obtained from marine sources are often considered promising materials with high potential in biomedicine [23]. As compared to biomaterials obtained from mammals, marine biomaterials are free from the risk of transmission of numerous diseases affecting human beings [24]. In addition, these biomaterials are often obtained as by-products of the fishing industry, and their use in biomedicine could contribute to providing this industry with increased economic value using an environmentally sustainable source [24,25]. One of the alternative biomaterials showing potential usefulness in tissue engineering is the squid gladius (SG). The SG is a soft tissue found as an internal structure in squids and other cephalopods devoid of a hard protective shell. Cephalopods, and squids in particular, are a burgeoning global food source with great commercial importance worldwide [26]. In addition to their importance as an essential food source, squids have been recently used for different purposes in biomedicine. Among other applications, the decellularized mantle has been used as a natural biomaterial in tissue engineering [27], whereas biocompatible biomaterials showing regenerative potential in wound healing were generated from the external skin [28], and the squid head cartilage has been applied to cartilage tissue engineering [29]. Regarding the SG, this structure has been used for the extraction and purification of several components, especially chitin and chitosan [30,31], but this is the first time that this structure has been used as a biomaterial.

Although the exact composition of the SG has not been elucidated in depth, the SG is known to contain high amounts of chitin and chitosan aminopolysaccharide polymers [32]. In fact, several authors have used this structure for the extraction and purification of chitin and chitosan using chemical extraction protocols [30,31]. Although different types of hydrogels have been generated from the extracts obtained from the SG, the use of the SG structure as a biomaterial itself has not been explored to date. The apparent physical properties of this structure, which provides resistance and elasticity to the animals and is very transparent, make this product an ideal candidate for corneal tissue engineering.

In the present work, we have analyzed the SG of a common species of squid to determine its potential application as a natural scaffold for use in cornea tissue engineering.

## 2. Results

### 2.1. Histological Analysis of Native SG (N-SG)

**Figure 1:**
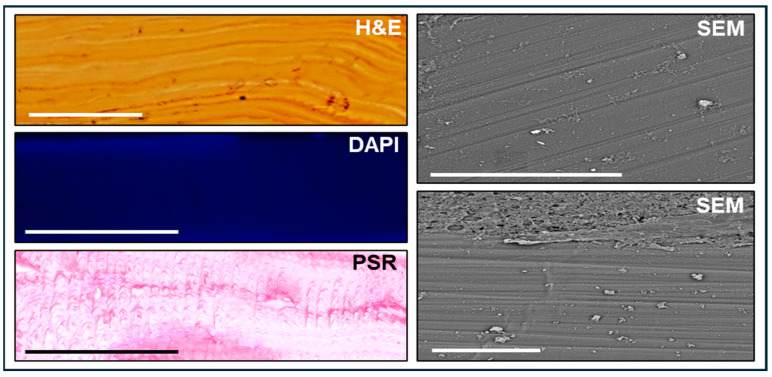
Histological analysis of the native squid gladius (N-SG) used in the present work. N-SG were analyzed using hematoxylin-eosin (H&E) to determine the internal structure of the biomaterial using transversal sections, DAPI, to identify cell nuclei, picrosirius red (PSR), to stain collagen fibers and scanning electron microscopy (SEM), to analyze the structure of the disk surface using two different orientations of the material. Scale bar: 50 µm.

Analysis of the native SG disks obtained from squid specimens of the species *Illex coindetii* using hematoxylin and eosin (H&E) staining (Figure 1) revealed that this biomaterial consisted of a dense, homogeneous layer of material consisting of numerous lamellae in which blood vessels, glands or other tissue specializations were not found. Instead, this biomaterial was formed by a regular structure with very few differentiations between the top and the bottom areas. When the presence of squid cells was evaluated using DAPI fluorescent analysis, we found that N-SG was an acellular structure completely devoid of cells. In addition, we wanted to determine if the main constituent of the N-SG was collagen, using picrosirius red histochemistry (PSR). Results showed that collagen was scarce in this structure. Finally, our surface analysis using scanning electron microscopy (SEM) revealed that N-SG had a very smooth surface with numerous parallel bands that may correspond to growth lines. When the internal structure and special distribution of the material were analyzed, results showed that the N-SG mainly consisted of numerous parallel layers of dense material with low porosity (Figure 1).

### 2.2. In Vivo Biocompatibility Analysis of N-SG Biomaterials

**Figure 2:**
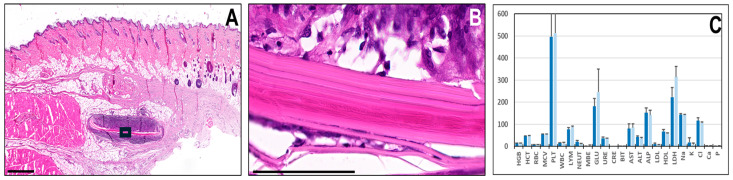
Analysis of biocompatibility in vivo of the native squid gladius (N-SG) grafted subcutaneously in laboratory animals. (**A**) Histological analysis of the implant site using H&E staining (scale bar: 1000 µm); (**B**) Higher magnification image of the area highlighted with a black square in A (scale bar: 100 µm); (**C**) Analysis of hematological and biochemical parameters in blood of animals grafted with N-SG, with results found in animals shown in dark blue and normal values shown in light blue. HGB: hemoglobin (g/dL), HCT: hematocrit (%), RBC: red blood cells (106/μL^−1^), MCV: mean cell volume (fL), PLT: platelets (105/μL^−1^), WBC: white blood cells (103/μL^−1^), LYM: percentage of lymphocytes (%), NEU: percentage of neutrophils (%), MBE: percentage of monocytes–basophils–eosinophils (%), GLU: glucose (mg/dL), URE: urea (mg/dL), CRE: creatinine (mg/dL), BIT: total bilirubin (µmol/L), AST: aspartate transaminase (U/L), ALT: alanine transaminase (U/L), ALP: alkaline phosphatase (U/L), LDL: low-density lipoprotein (mg/dL), HDL: high-density lipoprotein (mg/dL), LDH: lactate dehydrogenase (U/L), Na: sodium (mmol/L), K: potassium (mmol/L), Cl: chlorine (mmol/L), Ca: calcium (mmol/L), P: phosphorous (mmol/L).

In order to evaluate the in vivo biocompatibility of N-SG, this biomaterial was grafted subcutaneously in laboratory rats. As shown in Figure 2A,B, histological analysis of the grafting site revealed that the biomaterial was still detectable after 30 days of the implant. The histological structure of the N-SG showed that this biomaterial was surrounded by giant cells, macrophages and other types of white blood cells, which formed a pseudo capsule around the biomaterial. Apparently, cells were progressively invading the superficial layers of the biomaterial, which were detached from the core biomaterial and probably degraded. No signs of necrosis, tumorigenesis, hemorrhage or other complications were detected, and all tissues surrounding this pseudo capsule were compatible with normal tissues.

Furthermore, the analysis of relevant hematological and biochemical parameters in the blood of the animals grafted with the biomaterial was devoid of any detectable alteration, with all parameters within a normal range (Figure 2C).

### 2.3. Histological Analysis of Cellularized SG (C-SG)

**Figure 3:**
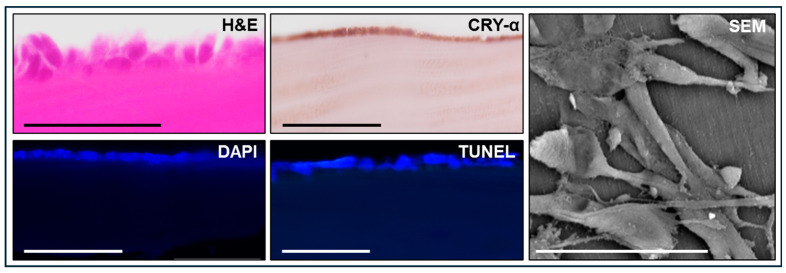
Histological analysis of the cellularized squid gladius (C-SG) used in the present work. C-SG were analyzed using hematoxylin-eosin (H&E) and scanning electron microscopy (SEM) to determine the structure of the C-SG product, DAPI, to identify cell nuclei, immunohistochemistry for crystallin alpha (CRY-α) and TUNEL to evaluate cell viability. Scale bar: 50 µm.

When corneal epithelial cells were cultured on the surface of these biomaterials to generate a cellularized SG (C-SG) that could resemble a bioartificial cornea generated by tissue engineering, we found that corneal epithelial cells were able to attach to the surface of this biomaterial and to form an epithelial layer partially resembling the corneal epithelium, although with only 2–3 cell strata (Figure 3). The presence of cells at the surface of the biomaterial was confirmed by DAPI. Furthermore, we found that cells were viable, and an apoptosis process was not ongoing in these cells, as determined by TUNEL. Then, we evaluated the expression of crystallin alpha (CRY-α), a relevant marker of corneal epithelium differentiation, by the epithelial layer of C-SG using immunohistochemistry. Results revealed that these cells retained their positivity for this marker in C-SG. In addition, the SEM analysis of C-SG showed that corneal epithelial cells attached to the surface of the biomaterial displayed an elongated spindle-shaped or star-like morphology.

### 2.4. Analysis of Biomechanical Properties of N-SG, C-SG and Human Native Cornea

**Figure 4:**
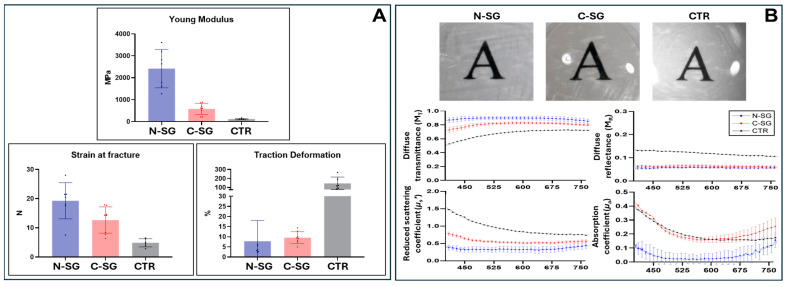
Analysis of the biomechanical (panel (**A**)) and optical properties (panel (**B**)) of the native squid gladius (N-SG), cellularized squid gladius (C-SG) and native cornea (CTR). For the biomechanical properties, the Young modulus, strain at fracture and traction deformation values are shown. For the optical properties, the top figures show the gross transparency of each sample allocated on a printed black letter, and the bottom figures correspond to the analysis of diffuse transmittance (M_T_), diffuse reflectance (M_R_), reduced scattering (*μ_s_*’) and absorption coefficient (*μ_a_*) for each type of sample.

When the biomechanical properties of the different samples analyzed in the present work were evaluated, we found significant differences among sample types (Figure 4A and Table 1). First, we found that the Young modulus significantly differed among the three types of samples analyzed here (N-SG, C-SG and CTR) (ANOVA *p*-value < 0.0001). When samples were compared pairwise, we found that the Young modulus of N-SG was significantly higher than that of C-SG and native human corneas used as controls (CTR), with N-SG showing more than 20-fold the values found in CTR. However, C-SG were comparable to CTR, with differences between both types of samples being statistically non-significant. Then, we analyzed the strain at fracture, and we found that the three sample types were statistically different for this parameter (ANOVA *p* = 0.0004). For the pairwise comparisons, the results of these analyses revealed significant differences between CTR and SG samples, with N-SG showing the highest values, and CTR showing the lowest values, although N-SG and C-SG were not significantly different. Finally, the traction deformation was evaluated in the three types of samples, and we again found statistical differences among the three sample types (ANOVA *p* = 0.0001). When samples were compared pairwise, we found that N-SG were comparable to C-SG (Mann-Whitney *p* > 0.05), and both samples were significantly lower than CTR.

### 2.5. Analysis of Optical Properties of N-SG, C-SG and Human Native Cornea

On the one hand, the gross analysis of transparency showed that the three samples evaluated in this work (N-SG, C-SG and CTR) were apparently transparent, allowing the printed letters to be readable through these biomaterials (Figure 4B). In addition, we analyzed the optical properties of each type of sample using Inverse Adding and Doubling methods. Results showed that the spectral diffuse transmittance presented similar optical behavior, but the values were significantly different among the three types of samples (N-SG, C-SG and CTR) (*p* < 0.0001 for the Kruskal-Wallis test). N-SG showed significant differences with C-SG and with CTR, and C-SG showed significant differences with CTR for the pairwise comparisons (*p* < 0.001 for the Mann-Whitney test for all pairwise comparisons) (Figure 4B).

The three key parameters that could be associated with the diffuse transmittance, including the diffuse reflectance, the scattering and the absorption coefficients, were analyzed in the study. Significant differences among the three types of samples were found for these three parameters (*p* < 0.0001 for the Kruskal-Wallis test). As shown in Figure 4B, CTR corneas showed the highest values of diffuse reflectance, scattering and absorption (*p* < 0.001 for the comparison of CTR vs. N-SG and CTR vs. C-NG), whereas N-SG showed the lowest results of these three parameters (*p* < 0.001 for the comparison of N-SG vs. CTR and N-SG vs. C-NG). Scattering was the parameter that showed the greatest influence on transmittance.

## 3. Discussion

In the present work, we have generated SG biomaterials obtained from a natural source that proved potential usefulness in cornea tissue engineering. When the structure of N-SG was analyzed, we found that this biomaterial was homogeneous, formed by abundant parallel lamellae of material, and apparently contained no host cells or specialized structures. This uniform structure makes N-GS a promising biomaterial for use in cornea tissue engineering. A crucial step in the development of novel biomaterials and tissue substitutes for clinical use is in vivo evaluation in animal models. In fact, in vivo evaluation and characterization are among the requirements of national medicine agencies regulating advanced therapies and the authorization of medicinal products [16]. In the case of the novel SG biomaterials described in the present work, these products were implanted subcutaneously in laboratory animals to determine the potential of these products to integrate into the host without generating any side effects. At the systemic level, results show that the main parameters analyzed in the blood of the grafted animals were within normal ranges, suggesting that the implant was safe and did not significantly alter the core metabolic functions of these animals. Furthermore, the histological analysis of the local implant site suggests that SG was biocompatible, although an initial phase of local inflammation restricted to the implant site was detected, with no other alterations such as rejection, tumorigenesis, necrosis, infection or other possible complications. These results are in agreement with previous studies suggesting that chitosan-based biomaterials grafted in laboratory animals could exert a mild to moderate chronic inflammatory reaction that tends to disappear with time and does not compromise biodegradation and biocompatibility [33]. Although future studies should be carried out to evaluate its behavior in vivo after longer periods of time, our results suggest that SG biomaterials could be biocompatible when grafted in vivo in laboratory animals.

Once we evaluated the structure and biocompatibility of N-SG, we recellularized this biomaterial with corneal cells to generate a corneal substitute by tissue engineering. Results showed that this biomaterial can be efficiently used as a scaffold able to support corneal cell attachment and growth without any detectable alterations. In addition, cells kept their typical elongated morphology and showed a negative staining signal for TUNEL, suggesting that cell viability and survival were not affected after the SG biomaterial [34,35]. However, the epithelial layer generated on top of the SG biomaterial was not fully differentiated, contained a low number of cell layers and was devoid of some of the fine features displayed by the native cornea epithelium. Previous reports demonstrated that bioartificial tissues generated in the laboratory are undifferentiated, and obtaining a fully functional and fully differentiated epithelium requires longer incubation times, the use of conditioning culture media and in vivo grafting in laboratory animals [36].

In general, we found that bioartificial corneas retained the fine structure of the SG biomaterial and supported cell development, which are essential requirements of novel biomaterials for use in tissue engineering [16]. Although future studies should determine the capability of SG to support cell growth and differentiation using other types of corneal cells, such as stromal keratocytes, corneal endothelial cells and human limbal cells, these results support the ex vivo biocompatibility of these marine-derived biomaterials and their potential use in tissue engineering. Even though the material resulted in low porosity, and we were not able to find cell diffusion and growth within the biomaterial at the time studied, our results support the use of corneal cells on the surface of these biomaterials. Future studies should determine the specific applications of these products.

Biomechanical properties are crucial requirements that biomaterials intended for use in tissue engineering must fulfill. In the case of the human cornea, biomaterials should resemble the viscoelasticity, resistance and elasticity of the native cornea, with a stable and resilient behavior [16,37]. Strikingly, our analyses revealed that N-SG were significantly superior to the native cornea in terms of Young modulus and strain at fracture and significantly lower for traction deformation. These results imply that N-SG could be stiffer but less elastic than the human cornea, which could hinder their clinical use in patients with corneal damage. As the cornea is considered to be a viscoelastic tissue [38], an equilibrium between resistance and elasticity is necessary for these biomaterials to be ideal for use in cornea tissue engineering. An interesting finding of our work was the fact that recellularization resulted in a reduction of the stiffness, with a decrease in the Young modulus. In consequence, C-SG could fulfill the biomechanical requirements of biomaterials used in corneal tissue engineering, as reported for other natural biomaterials [37]. An intriguing finding of our study is the values of the biomechanical parameters found in native corneas, which are higher than those normally reported by the literature [38,39]. In this regard, it is important to note that no consensus exists regarding the biomechanical properties of the native cornea, and several reports found very different results, ranging from almost 0 to more than 50 MPa, according to a recent review [40]. Most likely, these differences are related to the testing conditions and the methods used to evaluate these parameters, which are highly variable among reports, as previously suggested [39].

An important parameter for biomaterials used in tissue engineering is their optical behavior. In general, SG biomaterials showed very high levels of transparency, as determined by their diffuse transmittance, and very low reflectance, scattering and absorption, parameters that could reduce the tissue transmittance [28,30]. In this regard, it has been demonstrated that the high transparency of the human native cornea derives from the fine arrangement of collagen fibrils forming regularly packed lamellae able to reduce light scattering [41]. Although N-SG contained very few amounts of collagen, the arrangement of the components of this material in a regular and well-organized 3D disposition may also explain its transparency properties. Interestingly, our analysis revealed that light transmittance was higher in N-SG and C-SG than in CTR. Although further analyses are needed to explain the molecular bases of this finding, we might hypothesize that both the narrow packaging of the SG lamellae and the lack of cells could improve the transmission of light through SG. In fact, it has been demonstrated that corneal stromal cells may reduce light transmission, and these cells must synthesize crystallins as specialized proteins able to counteract this phenomenon [42]. Interestingly, recellularization of N-SG to generate C-SG was associated with a decrease in light transmittance, although values remained significantly higher than CTR. In general, these results confirm the idoneousness of these novel biomaterials to support corneal tissue engineering from an optical standpoint.

An important parameter in cornea tissue engineering is the thickness of the products generated by tissue engineering. In general, it is well known that the thickness of the human native cornea is 0.5–0.6 mm in the central cornea, with an increase in the peripheral cornea [43]. In our study, however, the SG disks were about 1 mm thick. In consequence, the results of the biomechanical and optical analyses should be taken with caution since the use of biomaterials with different thicknesses may modify these results. The fact that these SG can be trimmed to desired shapes and thicknesses may offer an additional advantage to the use of these biomaterials.

Although the novel products generated in the present work should still demonstrate usefulness in a real clinical scenario, our results support the idea that these SG should be considered good candidates for cornea tissue engineering. These biomaterials can be obtained from a natural marine source and have been shown to have good biological and physical properties for use as biological scaffolds in tissue engineering.

## 4. Materials and Methods

### 4.1. Preparation of the SG Biomaterials

**Figure 5:**
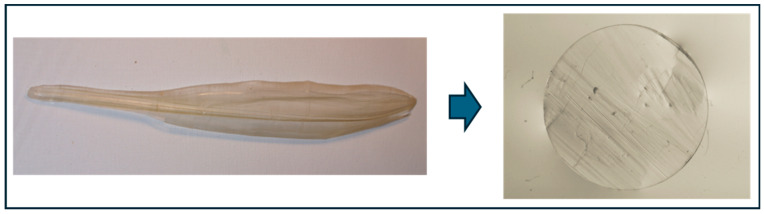
Gross images of the native squid gladius (N-SG) used in the present work. The left image shows the macroscopic appearance of the whole SG after extraction from an *Illex coindetii* specimen, whereas the image to the right shows an illustrative N-SG disk.

The squid internal gladius was obtained from 10 specimens of *Illex coindetii* purchased at the local fish market. This structure was carefully dissected and washed several times in sterile PBS (Merck, Burlington, MA, USA). Disks approximately 8 mm in diameter and 1 mm thick were obtained with a biopsy punch (Kay Medical, Seki, Japan) from each SG (Figure 5). These samples were considered as native SG biomaterials (N-SG).

In order to remove any possible rests of inorganic substances and to prepare the disks for cell culturing, samples were incubated in a solution containing 50% formic acid and 20% sodium citrate for 24 h, followed by three washes in distilled water as previously suggested [44].

### 4.2. Generation of a Bioengineered Corneal Substitute by Tissue Engineering

SG were recellularized using corneal epithelial cells to generate C-SG. In brief, disks were washed in PBS, and 200,000 limbal epithelial cells of the SIRC cell line (Statens Seruminstitut Rabbit Cornea) were subcultured on top of each disk. To promote call attachment, these cells were previously resuspended in 50 µL of DMEM medium (Dulbecco’s modified Eagle’s medium, Merck, Darmstadt, Germany) supplemented with 10% of fetal bovine serum (Merck), and this volume of medium was carefully distributed on all the surface of the disks. After an incubation period of 2 h, 1 mL of culture medium was added to each sample. C-SG were incubated for 4 days in a cell incubator at 37 °C with 5% CO_2_ using standard cell culture conditions to allow cells to attach and grow on the disk surface.

### 4.3. Analysis of Biomechanical Properties

Biomechanical properties of N-SG, C-SG and control native corneas (CTR) were performed using a biomechanical analyzer Instron 5943 (Norwood, MA, USA) with a BlueHill 3 Material Testing software (version 3). Each sample was analyzed using a continuous tension stress force with a constant strain rate of 5 mm/min until the material was fractured. In each case, the Young modulus was quantified by determining the tangent modulus of the linear portion of the stress-strain curve, whereas the strain at fracture was calculated by determining the point of the curve where the fracture occurred, and the traction deformation was calculated as the percentage of elongation that the biomaterial reached before fracturing. Six samples of each type were analyzed (*n* = 6).

### 4.4. Analysis of Optical Properties

To determine the optical properties of the N-SG, C-SG and control native corneas (CTR) specimens, the Inverse Adding-doubling (IAD) method was employed [45]. The experimental values of the total diffuse reflectance (M_R_) and transmittance (M_T_) used in the IAD method were determined by measuring the total diffuse reflection and transmission using a customized double-integrating sphere setup [46]. Measurements were performed in the visible wavelength range of 400–780 nm using a white light source (240–1100 nm, Thorlbas, Dortmund, Germany) connected to the double integrating sphere system by an optic fiber (M92L01, Φ = 200 µm, 0.22 NA) as a light source. The N-SG, C-SG and CTR samples were sandwiched between two optical borosilicate glass slides (1.1 mm thickness).

From the diffuse reflectance (M_R_) and diffuse transmittance (M_T_) measurements, the absorption coefficient (μ_a_) and the reduced scattering coefficient (μ_s_′ = μ_s_ (1 − g)) were determined using the IAD algorithm available at https://omlc.org/software/iad/ (accessed on 26 November 2024). With the coefficient values obtained numerically, we calculated new values of M_R_ and M_T_ that were compared with the measured ones. The value of g was assumed to be 0.98. According to the IAD algorithm [45], this process was iterated until the calculated and experimentally obtained values of M_R_ and M_T_ were within a specified tolerance (for the current study, the tolerance default value was set to 0.01%).

To solve the inverse problem, the knowledge of the refractive index of the sample is required. Changes in the refractive index over the range of wavelengths employed in the present study were assumed negligible, and therefore, we consider the refractive index n = 1.44 as the mean value for the visible range, as previously reported [47].

### 4.5. In Vivo Evaluation in Laboratory Animals

To determine the in vivo biocompatibility of the SG biomaterials generated in the present work, SG disks were subcutaneously implanted in laboratory animals. Five 12-week-old male Wistar rats were deeply anesthetized with ketamine and acepromazine (Boehringer Ingelheim, Ingelheim am Rhein, Germany), and a subcutaneous pouch was generated at the interscapular area of each animal. A disk of SG biomaterial was then allocated to the subcutaneous pouch, and the surgical injury was repaired using surgical staples. Animals were kept for 1 month in the animal facility with free access to water and chow. After this time, all animals were euthanatized under general anesthesia, and the local site of the implant was surgically extracted in toto for histological analysis.

In addition, 1 mL of peripheral blood was extracted at the moment of euthanasia for the analysis of key hematological and biochemical parameters in the blood of the animals grafted with the biomaterials. For this purpose, blood samples were analyzed with a Sysmex KX-21N automatic analyzer and a Cobas c311 analyzer (Roche, Basel, Switzerland).

### 4.6. Histological Analysis

For histological analysis, samples were fixed in 4% neutral formaldehyde, dehydrated in ethanol, cleared in xylene, and embedded in paraffin using routine histological protocols. Four µm-thick tissue sections were obtained using a microtome, dewaxed, and rehydrated. These samples were then stained with hematoxylin and eosin (H&E) following standard laboratory protocols. In brief, tissue sections were incubated for 3 min in hematoxylin, differentiated in tap water for 5 min and incubated in eosin for 1 min. Slides were then dehydrated and coverslipped using a histological mounting medium. All these reagents were obtained from Panreac AppliChem (Barcelona, Spain). Analysis of collagen fibers was carried out using picrosirius red histochemistry (PSR), as previously reported. Briefly, slides were incubated in sirius red F3B solution for 30 min and counterstained with Harris’s hematoxylin for 5 min. Expression of the corneal marker crystallin alpha (CRY-α) was assessed by indirect immunohistochemistry using specific primary antibodies anti-CRY-αA (Santa Cruz Biotechnology, Santa Cruz, CA, USA). In this case, deparaffinized tissue sections were incubated in citrate buffer pH 6.0 for 25 min at 95 °C for antigen retrieval, followed by prehybridization with normal horse serum (Vector Laboratories, Burlingame, CA, USA) and incubation with the primary antibody at a dilution of 1:100. Samples were then washed in PBS, incubated in ready-to-use anti-mouse secondary antibodies (Vector Laboratories), and incubated in diaminobenzidine (DAB) substrate kit (Vector Laboratories). Tissues were then briefly counterstained with Harris hematoxylin (Thermo Fisher Scientific, Waltham, MA, USA) and coverslipped. Images of samples stained with H&E, PSR and CRY-α were obtained using a Pannoramic^®^ Flash Desk DW histological scanner (3DHISTECH, Budapest, Hungary).

To identify cells on the surface of C-SG, tissue sections were stained with 40,6-diamidino2-phenylindole (DAPI), coverslipped and examined with a Nikon Eclipse i90 fluorescent microscope. For the analysis of surface structure, disks were fixed in 2.5% glutaraldehyde, dehydrated in acetone, critical point dried and sputter-coated with gold, following routine laboratory procedures. These samples were then evaluated using a FEI Quanta 200 scanning electron microscope (FEI, Eindhoven, The Netherlands). Terminal deoxynucleotidyl-transferase dUTP Nick-End Labeling (TUNEL) assays were used to analyze cell viability by identifying apoptotic cells. In brief, tissue sections were treated with a 20 μg/mL solution of proteinase K for 5 min, incubated in a DeadEnd™ Fluorometric TUNEL System (Promega, Madison, WI, USA) reaction solution for 1 h at 37 °C, washed and stained with DAPI. Positive and negative controls were used.

## Figures and Tables

**Table 1:** Analysis of biomechanical properties of the native squid gladius (N-SG), cellularized squid gladius (C-SG) and control corneas (CTR).

	Young Modulus (MPa)	Strain at Fracture (N)	Traction Deformation (%)
N-SG	2383.25 ± 953.83	19.53 ± 6.74	4.27 ± 1.55
C-SG	580.66 ± 301.24	13.09 ± 4.93	10.14 ± 3.28
CTR	114.8 ± 28.57	4.87 ± 1.4	146.04 ± 70.09
N-SG vs. C-SG	0.0002 *	0.0894	0.9711
N-SG vs. CTR	<0.0001 *	0.0003 *	0.0002 *
C-SG vs. CTR	0.3674	0.0273 *	0.0002 *
N-SG vs. C-SG vs. CTR	<0.0001 *	0.0004 *	0.0001 *

* Differences are statistically significant.

## Data Availability

The original data presented in the study are openly available in Zenodo at https://doi.org/10.5281/zenodo.14210696.
